# Research on Detection and Location of Fluid-Filled Pipeline Leakage Based on Acoustic Emission Technology

**DOI:** 10.3390/s18113628

**Published:** 2018-10-25

**Authors:** Shengshan Pan, Zhengdan Xu, Dongsheng Li, Dang Lu

**Affiliations:** 1 State Key Laboratory of Coastal and Offshore Engineering, Dalian University of Technology, Dalian 116024, China; pssbu@dlut.edu.cn (S.P.); icve2008@dlut.edu.cn (Z.X.); ludangcumt@163.com (D.L.); 2 School of Civil Engineering, Dalian University of Technology, Dalian 116024, China

**Keywords:** fluid-filled pipe, acoustic emission, support vector machine, leak location

## Abstract

Because of the inconvenience of installing sensors in a buried pipeline, an acoustic emission sensor is initially proposed for collecting and analyzing leakage signals inside the pipeline. Four operating conditions of a fluid-filled pipeline are established and a support vector machine (SVM) method is used to accurately classify the leakage condition of the pipeline. Wavelet decomposition and empirical mode decomposition (EMD) methods are initially used in denoising these signals to address the problem in which original leakage acoustic emission signals contain too much noise. Signals with more information and energy are then reconstructed. The time-delay estimation method is finally used to accurately locate the leakage source in the pipeline. The results show that by using SVM, wavelet decomposition and EMD methods, leakage detection in a liquid-filled pipe with built-in acoustic emission sensors is effective and accurate and provides a reference value for real-time online monitoring of pipeline operational status with broad application prospects.

## 1. Introduction

Pipeline transport has become the fifth largest transportation tool in modern society and offers great advantages in transporting fluid media (e.g., oil, natural gas, water). Pipelines in China have spread across large areas of land and sea and are an important approach to boosting regional economic ties. However, existing pipelines are often vulnerable to damage due to their long service life, corrosion due to aging and external load impact, leading to major accidents. Therefore, an effective structural health monitoring is important for pipeline engineering. 

At present, pipeline damage monitoring methods include remote field eddy current testing, ultrasonic guided waves, vibration sensors and modified ML pre-filters, distributed fiber Bragg grating sensors and others [1,2,3,4]. When a liquid-filled pipe is damaged, the fluid generates a stress wave near the leak that rapidly propagates along the wall. Hence, an acoustic emission (AE) technology is used to collect and analyze the signal and to detect the leak in the pipeline. The AE detection method for pipelines can achieve real-time, dynamic monitoring. It has been applied to detect leakage in oil and gas pipelines. Local and international scholars have conducted extensive research in this field. To improve the location accuracy of pipeline leakage sources, Wang Yongqing et al. [5] used the wavelet packet and cross correlation theories to investigate location detection. On the basis of the propagation theory of guided waves in pipes, Jingpin Jiao et al. [6] proposed a specific model of AE signal propagation in pipelines and introduced a new method to determine the leakage source. Li Zhenlin [7] proposed a novel leakage detection scheme based on kernel principal component analysis (kernel PCA) and the support vector machine (SVM) classifier for the recognition of the leakage level. Uncontrolled noise interference is an important factor that affects the accuracy of leakage location. Therefore, numerous scholars have started to focus on the data analysis and noise processing of AE signals from pipeline leakage and some research results have been obtained. For instance, through empirical mode decomposition (EMD) [8,9,10], the AE signal is initially decomposed and then a useful signal with noticeable signal features and high energy is reconstructed. Thereafter, leakage location is detected based on the reconstructed signal and accuracy is significantly improved. In addition, analysis methods based on wavelet theory [11,12] and HHT transform [13] are used in the decomposition and extraction of AE signals, which have certain effects on denoising. Gao Y et al. [14] used the generalized cross correlation (GCC) methods to find the leak location.

In current research, Shama et al. [15] simulated a section of underwater oil pipeline to study the possibility of using AE technology to monitor leakage in a submarine oil pipeline. The researchers set up various defects and leaks with different flow rates in the pipelines. The results show that AE can monitor and identify leakage in underwater oil pipelines. To address the inconvenience of leak measurement in long-distance buried pipelines, Xu et al. [16] designed a guide rod that can be inserted into the soil. This rod extends directly to the surface of the pipeline. The AE information transmitted by the guide rod is used to locate the damaged area. For pipeline leakage with high-pressure gas, Mostafapour et al. [17,18] deduced the radial displacement of pipeline vibration in AE wave propagation and compared the measured signal spectrum with numerical simulation calculation, proving that the AE stress wave can theoretically be used in pipeline leakage detection.

This paper attempts to solve two problems related to leakage monitoring in buried liquid-filled pipes. The first one is installing AE sensors. This study attempts to locate the AE sensors installed inside a pipeline to collect leakage signals and to verify the effectiveness of the method used. This provides a basis for subsequent built-in self-capacitive AE sensors to monitor the damage of fluid-filled pipelines. The second one is identifying leakage types and locations. A support vector machine (SVM) method is used for leakage detection in a liquid-filled pipe with a built-in AE sensor and then methods based on wavelet decomposition and EMD are utilized to effectively denoise the leakage signal. Moreover, the time-delay estimation method is employed to accurately locate the leakage source in the pipeline.

## 2. SVM

An SVM is an algorithm developed on the basis of a statistical theory. As a powerful and practical learning machine, it is widely used in the field of pattern recognition, including face recognition, speech recognition, text classification, regression prediction and accident detection. The SVM mainly aims to obtain a maximal category boundary by minimizing structural risk. Therefore, an optimal decision boundary function is established [19].

### 2.1. Optimal Hyperplane

**Figure 1:**
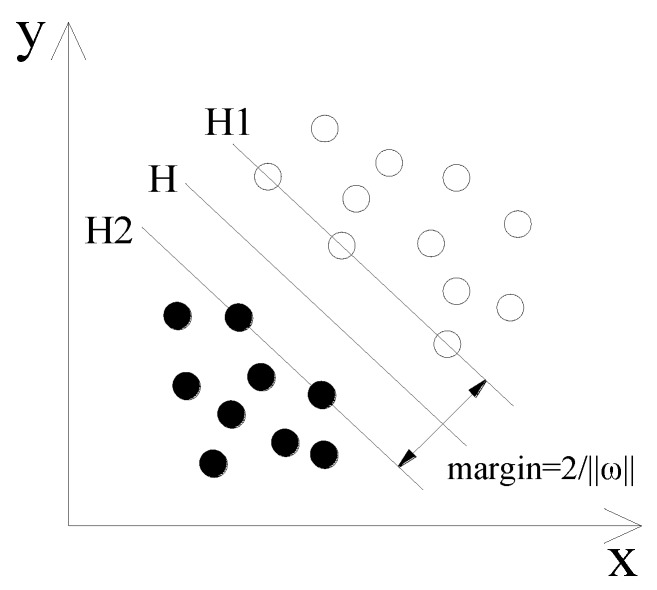
Optimal separating hyperplane in the support vector machine (SVM).

In Figure 1, the main idea of an SVM is explained in two dimensions. The hollow ○ and the solid ● are used to simulate two different samples and H is the classification plane. H1 and H2 pass through the sample data closest to H and parallel to the H plane. Their span is regarded as the classification margin. As shown in the figure, the optimal hyperplane has two main functions, namely (1) accurately classifying the training samples to ensure that the classification achieves the highest accuracy and (2) maximizing the classification interval and minimizing the confidence range of the extension community to ensure the lowest risk rate.

### 2.2. Selection of Kernel Functions

In the SVM, according to specific engineering problems, the corresponding kernel functions can be selected, which not only increases the computing power of the algorithm but also improves its accuracy. Four types of most frequently used kernel functions are as follows:

(1) Gaussian radial basis kernel (RBF) kernel function

(1) $$K\left(X_{l},X\right)=\exp\left(-g\cdot {\left\|X_{l}-X\right\|}^{2}\right),g>0\;$$

where ‖‖ is the norm in Euclidean space, g is the parameter representing the kernel width, $K\left(X_{l},X\right)$ is the kernel function, $X_{l}$ represents the $l_{th}$ training sample, and X is the characteristic values of the test sample.

(2) Linear kernel function

(2) $$K\left(X_{l},X\right)=X\cdot X_{l}\;$$

(3) Polynomial kernel function

(3) $$K\left(X_{l},X\right)={\left[k\left(X\cdot X_{l}\right)+m\right]}^{d}\;$$

where k is the slope of the polynomial, m is the intercept and d is the power.

(4) Sigmoid kernel function

(4) $$K\left(X_{l},X\right)=\tanh\left[\kappa \left(X\cdot X_{l}\right)-\delta \right]\;$$

According to the SVM principle, the characteristic parameters of the AE signal for pipeline leakage are numerous and unordered and their difference is not noticeable. When a hyperplane cannot effectively separate the samples, the Gaussian RBF kernel function, which is the most widely-used function, is feasible. The variable g represents the kernel width of the RBF kernel function, which determines the complexity of the algorithm. A penalty factor c determines the performance of the SVM classifier. Therefore, (c,g) is used as a search optimization variable, which can be obtained using the grid search method [20] and corresponds to the maximal accuracy of the classification.

### 2.3. Acoustic Emission Technology

**Figure 2:**
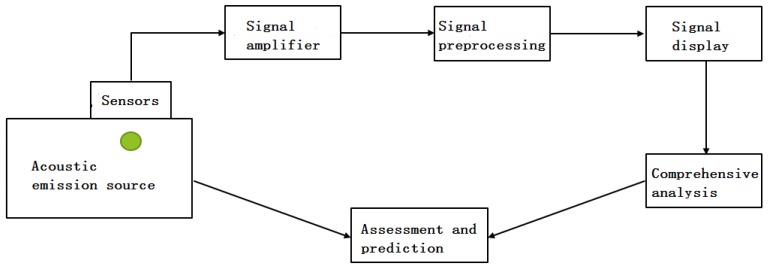
Schematic diagram of acoustic emission.

As shown in Figure 2, the AE analysis system includes three parts: a dedicated sensor for AE, a signal amplifier and a signal analysis system. The AE detection process includes: engineering material acting as a propagation medium to propagate the instantaneous elastic wave generated by the AE source to the surface of the material; an AE sensor coupled to the material’s surface receives a signal source and deforms; in order to obtain a digital signal that can be computed by a computer, the received mechanical signal is converted and amplified by a piezoelectric effect mechanism and is finally recorded in the form of a waveform; considering the material's characteristics, the collected AE characteristic parameters and the waveform information of the signal, the purpose of inferring the damage state of the AE source of the tested object is achieved.

## 3. Leakage Source Localization Based on Cross-Correlation Analysis

**Figure 3:**
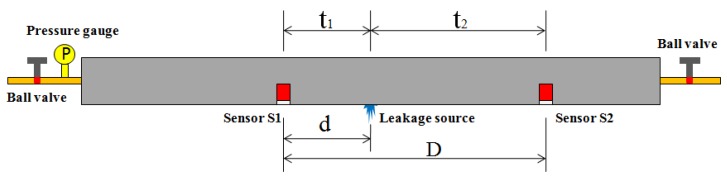
Schematic diagram for detection.

Leakage from a liquid-filled pipe produces a continuous AE signal. Leakage detection can be performed by collecting the leakage signal and analyzing related characteristics through AE equipment. The leakage location can be determined by installing a pair of sensors inside a pipe. Figure 3 shows the two sensors arranged on both sides of the leak.

Therefore, the positioning formula of the pipeline leakage source is as follows:

(5) $$d=\frac{D-\nu \cdot \Delta t}{2},\Delta t=t_{2}-t_{1}\;$$

The relevant parameters in the formula are shown in the figure, where ν is the propagation speed of the AE wave on the liquid-filled steel pipe. Therefore, obtaining two unknown quantities, namely, the speed ν and the time difference Δt, is the key to leak location. In this study, the propagation speed of the AE signal is calculated via the famous Nielsen–Hsu lead-cut experiment [21,22,23].

To obtain Δt, we use a time-delay estimation method based on cross correlation analysis, which is widely used in processing continuous AE signals. Assuming that the AE signals collected by the sensors on both sides are x(t) and y(t), the corresponding mathematical model is as follows:

(6) $$x\left(t\right)=s\left(t\right)+n_{1}\left(t\right),y\left(t\right)=\alpha s\left(t-\tau \right)+n_{2}\left(t\right)\;$$

where s(t) is the leakage source signal, α is the attenuation factor, τ is the delay time and $n_{1}\left(t\right),n_{2}\left(t\right)$ are the ambient noise.

Within a certain integral time *T*, the cross correlation formula is as follows:

(7) $${\hat{R}}_{xy}\left(\tau \right)=\frac{1}{T}\int_{0}^{T}\alpha s\left(t\right)s\left(t-\tau \right)dt\;$$

As shown in the cross correlation formula, the two AE signals are a function of the delay time τ. In the cross correlation diagram, the position of the peak point implies the appearance of the maximal correlation coefficient of the two signals and the corresponding $\tau_{0}$ is the signal time difference.

## 4. Wavelet Decomposition and EMD

By directly using the detected original signal waveform to calculate the cross correlation coefficient, a delay time with larger deviation is always obtained. Therefore, choosing an appropriate method to effectively denoise and optimize the original signal waveform is also key to the positioning accuracy. In the denoising of continuous AE signals, two typical methods are introduced in this section, namely, wavelet decomposition and EMD.

### 4.1. Wavelet Decomposition and Threshold Denoising

Wavelet analysis can be used to characterize the local time–frequency of nonstationary random signals with noise. This analysis not only realizes the function of removing a large amount of interference but also retains useful signal features [24]. The steps of wavelet threshold denoising are as follows:

(1) Wavelet decomposition of signals

**Figure 4:**
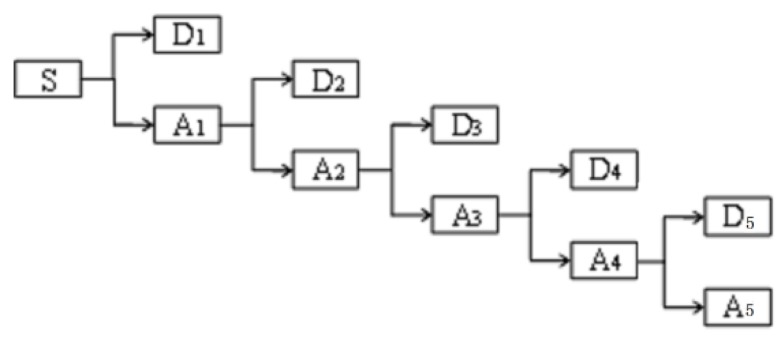
Four-layer wavelet decomposition process for signals.

Using a four-layer decomposition as an example, the wavelet decomposition process is graphically represented. As shown in Figure 4, A1–A5 represent the approximation functions of the layers, whereas D1–D5 represent the detail functions of the layers.

(2) Threshold quantization

After the original signal is decomposed by the wavelet, an appropriate threshold is used to quantize the high-frequency coefficients (D1–D5) of each layer to reduce the noise. The MATLAB software provides three threshold methods, that is, forced denoising, default threshold and given threshold.

(3) Signal reconstruction

Through threshold quantization, the decomposition coefficients $\left\{c_{j,n}\right\},\left\{d_{j,n}\right\}$ of each layer are used to reconstruct the signal using the MATLAB algorithm.

### 4.2. EMD

The EMD theory holds that any signal can be composed of a series of oscillating components with different scales, called intrinsic mode functions (IMFs). IMFs should meet two basic conditions as follows [25]:

(1) The total number of IMF extreme points is equal to the number of zeroes, or at most, there is a deviation.

(2) At any point in the IMF, the average value of the maximal and minimal points corresponding to the upper and lower envelopes, respectively, is equal to zero.

Based on these principles, the initial signal can be reconstructed from all decomposed IMF components and the final residual component $r_{n}\left(t\right)$, as follows:

(8) $$X\left(t\right)=\sum_{i=1}^{n}h_{i}\left(t\right)+r_{n}\left(t\right)\;$$

In this study, we initially use the EMD method to decompose the AE signal and obtain the IMFs and residual components and then we select a useful signal with evident features for reconstruction, thus achieving an effective denoising.

## 5. Experiment on Pipeline Leakage Detection Based on SVM

### 5.1. Experimental Scheme

**Figure 5:**
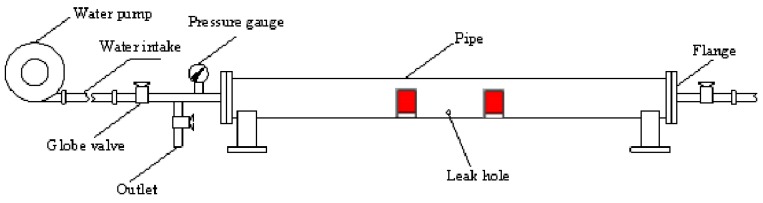
Schematic diagram of pipeline leakage test platform.

First, a simplified model for the operation of a liquid-filled pipe is constructed, as shown in Figure 5.

The AE testing equipment consists mainly of the following components: eight-channel Micro-II Digital AE System equipment produced by PAC for signal acquisition and analysis; SR40M AE sensor produced by ShengHua Technology Company used for collecting signals; an intelligent AE preamplifier that provides 20 dB, 40 dB and 60 dB gain; a bandwidth of 10 kHz–1.2 MHz that effectively amplifies the original AE signal; an attached device such as a computer screen or keyboard. Table 1 shows the parameter settings of the AE instruments.

**Figure 6:**
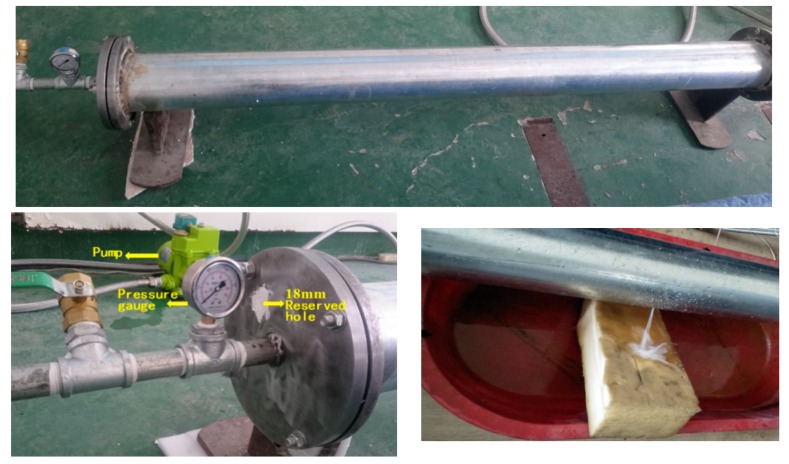
Laboratory model.

The main pipe used is a galvanized steel pipe with a length of 2 m, outer diameter of 165 mm and wall thickness of 4 mm. In the experiment, the water pressure in the pipe is regulated by adjusting the ball valve at both ends. The laboratory model is shown in Figure 6. The waterproof sensor is innovatively placed inside the pipe to detect the AE signal, to explore the transmission characteristics of the signal in the pipe when the pipe leaks and to explore the application of wireless sensors in the future. In addition, we propose the use of mobile robots for installation in practical application. Therefore, a hole with a diameter of 18 mm is reserved on the flange plate at both ends of the pipe for installation of the AE sensor. After installation, a leakage test is performed using a mud plug.

According to the literature, the peak frequency of a leakage signal in a pipeline is low; thus, the SR40M sensor of the narrowband low frequency is well matched. The related parameters of the sensor are listed in Table 2.

The processes of the leakage detection algorithm for liquid-filled pipes based on SVM are the following:

(1) Collect the characteristic parameters of the pipeline under different conditions using an AE instrument, select appropriate parameters and normalize them and set the normalization interval to [0, 1].

(2) Define labels for different states of the pipeline. For instance, set the pipeline standing state to 1, the normal operation state to 2 and the leakage state to 3.

(3) Divide all sample data into two parts, namely training and test sets and choose a suitable kernel function and corresponding parameters according to actual problems.

(4) Complete the testing process, build the classification prediction model using the determined kernel functions and parameters and establish the classification boundary function $f_{k}\left(x\right)$ to determine the optimal hyperplane.

(5) Use the optimal model constructed in Step (4) to perform diagnostic tests and input the characteristic parameters to complete the classification prediction of the test set.

### 5.2. Experimental Process and Result Analysis

A total of five conditions are set up in the experiment, as listed in Table 3.

According to the statistical mean and standard deviation of the collected data, several AE parameters with distinct discrimination and good stability were selected, such as energy, amplitude, ASL, average frequency and RMS.

The experimental conditions in Table 3 were combined to complete the following classification experiments.

(1) Two-way classification: resting (C1) and leakage (C3)

**Figure 7:**
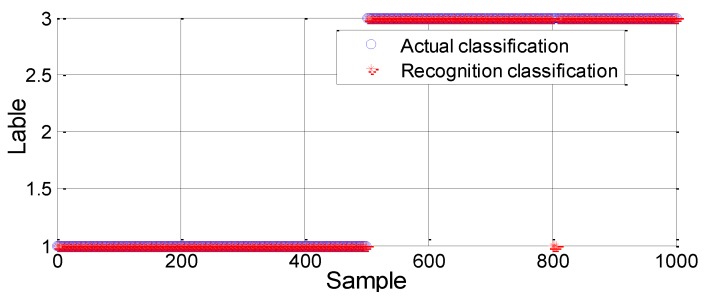
Classification results of the test sample (Two-way).

Pipeline resting and leakage are clearly different. Therefore, the linear kernel function can be used to train the SVM model. First, 100 datasets for each case are selected randomly to form the training set, which is used to train the classification model. Then, the remaining 500 samples for each case are imported into the classification model for test verification. The classification results are shown in Figure 7.

As shown in the results of Figure 7, there are seven erroneous judgments for the leakage conditions and the recognition accuracy rate is 99.3%. Therefore, based on statistical training data, the pipeline can be accurately detected using the SVM method in the event of a leak.

(2) Three-way classification: resting (C1), normal operation (C2) and leakage (C3)

First, 200 datasets of each case are randomly selected to form the training set. Then, the simplest linear kernel is used to train the model. The recognition results are not ideal, with a rate of accurate classification of only 87.5%.

**Figure 8:**
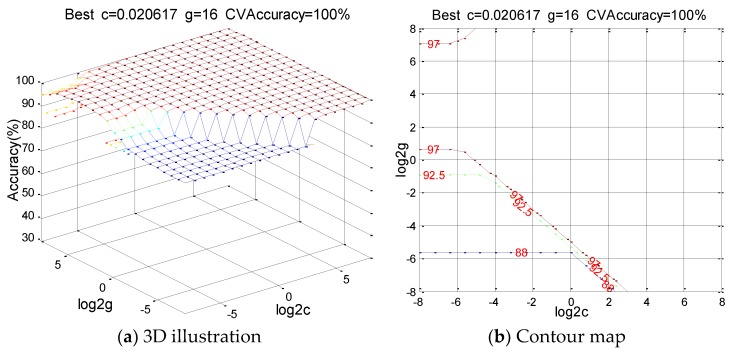
Results of parameter selection.

**Figure 9:**
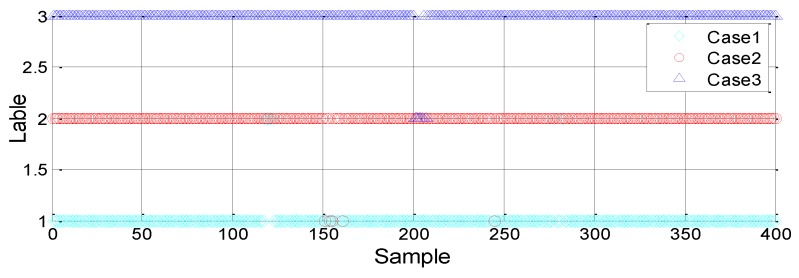
Test results based on the model with the RBF kernel function.

Therefore, the RBF kernel function is used to construct the SVM model. Meanwhile, the grid search method is used to find the corresponding penalty parameter c and kernel parameter g. The result of parameter selection is shown in Figure 8. The SVM model is established through the training set. By inputting the test sample into the model, each operating condition can be classified and determined and then the operating status of the pipeline can be detected. The test identification results are shown in Figure 9.

The test results of the RBF kernel function model for classification are quite accurate and the correct recognition rate for each case exceeds 98%. Unlike the recognition result of the linear kernel function, the integrated recognition accuracy rate is improved by 11%.

(3) Four-way classification: resting (C1), normal operation (C2), small pressure leakage (C4) and large pressure leakage (C5)

**Figure 10:**
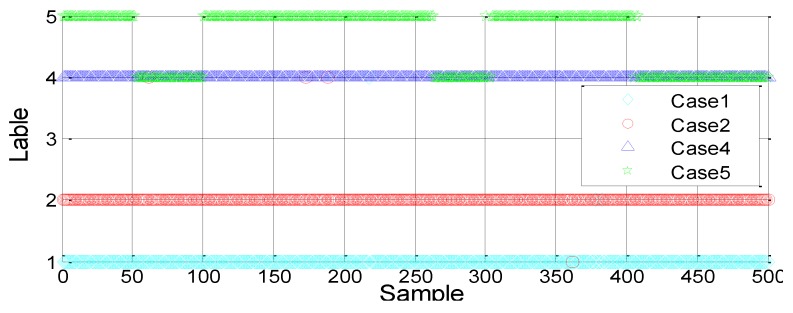
Classification results of the test sample using SVM (100 data for each case).

**Figure 11:**
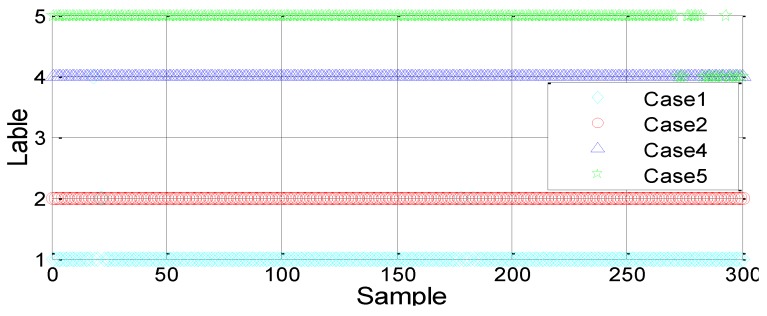
Classification results of the test sample using SVM (300 data for each case).

Generally, the larger the training dataset is, the more accurate the SVM model will be. A total of 100 and 300 data training models are selected for each case to investigate the effect of the sample number on the classification results. Figure 10 and Figure 11 show the results. The SVM model is also trained using the RBF kernel function.

By comparing the above two results, we can see that with the increase in training data samples, the classification accuracy rate improves significantly. Moreover, the four cases can be clearly divided and the number of undetermined cases is significantly reduced.

**Figure 12:**
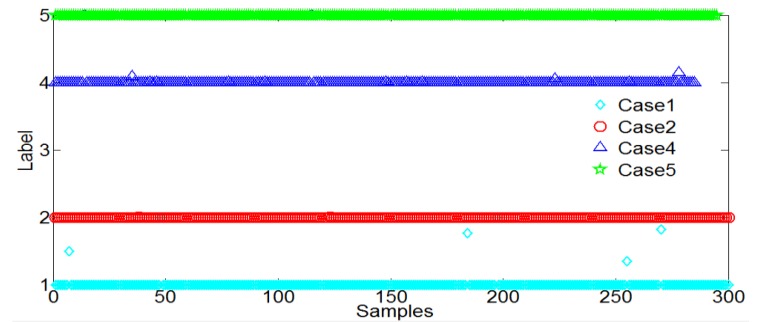
Classification results of the test sample using BP neural network.

In addition, the classification results of the proposed SVM method were also compared with the widely-used BP neural network. As shown in Figure 11 and Figure 12, the results of proposed SVM method nearly have the same accuracy with BP neural network.

## 6. Pipeline Leakage Location Based on Wavelet Decomposition and EMD

After detecting a leak in the pipeline, a method should be adopted to further determine the source of the damage. This section addresses the problem of locating the source of a pipeline leak. The propagation speed of AE waves on liquid-filled pipes was obtained through a lead-cutting experiment. The final calculated average wave speed is 3446 m/s. In practical applications, a database of wave speeds for different materials must be facilitated for the application of the method.

The pressure in the pipe is stable at 0.2 MPa and sensors S1 and S2 are placed on both sides of the leak in the pipe. Liquid leakage detection is performed after the pressure is stable and the signal is collected using an AE instrument. Moreover, five sensor placement conditions are set up for the study. The distances (d1–d2) from S1 and S2 to the hole are 400–600, 500–800, 650–800, 700–800 and 800–950 mm.

### 6.1. Wavelet Decomposition Denoising

According to the wavelet denoising theory, the wavelet denoising analysis of the leaking AE signal is performed using a DB6 wavelet, five-level decomposition and default threshold denoising.

**Figure 13:**
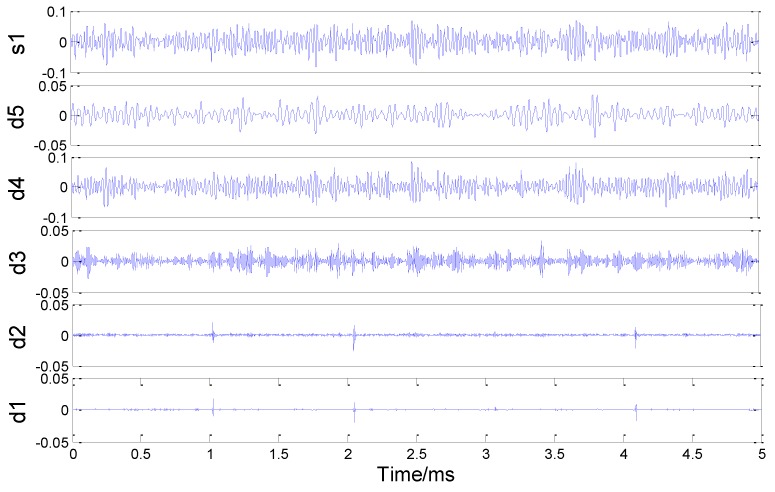
Details of signal wavelet decomposition.

**Figure 14:**
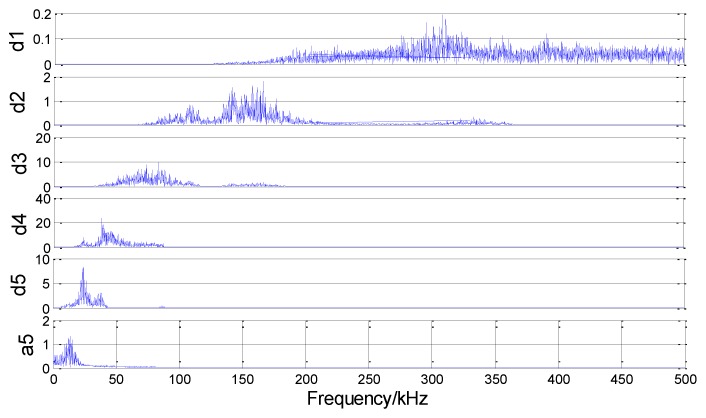
Spectrum of each layer after the signal wavelet decomposition.

The AE signal is decomposed into five scale wavelets, as shown in Figure 13, where a1–a5 represent the approximation functions (also called the low-frequency coefficients) decomposed in each layer and d1–d5 represent the detail functions (also called the high-frequency coefficients) decomposed in each layer. Noise signals usually exist in high-frequency coefficients. As shown in the figure, d1 and d2 have noticeable noise signal characteristics. Figure 14 shows the spectrum corresponding to the decomposition coefficient of each layer after the final wavelet decomposition. Using the default threshold method, a high-frequency coefficient for each layer is initially quantized with the threshold value, then the wavelet reconstruction is performed with a low-frequency coefficient a5 of the last layer and the signal after noise removal can be finally obtained.

**Figure 15:**
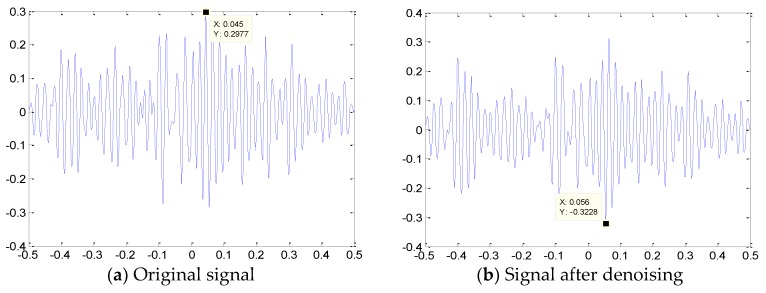
Cross-correlation function diagram of signals S1 and S2.

On the basis of the cross-correlation theory, the cross correlation function of the AE signal detected by the two sensors is obtained. The point of greatest cross correlation is the cross coordinate corresponding to the peak value in the function graph and, thus, the time difference Δt. Graphs (a) and (b) in Figure 15 represent the original signals and the cross correlation function diagram, respectively, after wavelet denoising of the two sensors. As shown in the graphs, the time difference between the original signals reaching the sensor is Δt=0.045 ms and the time difference obtained after noise reduction is Δt=0.056 ms. By substituting D, ν and Δt into Equation (5), we can calculate the distance between S1 and the leak point d1 to locate the leak source.

The experimental results of condition 1 are listed in Table 4. As presented in the table, the location accuracy of the leakage source evidently improves after the original signal is denoised by the wavelet. The results of other conditions are listed in Table 5. The positioning results at various distances are relatively accurate and the relative errors are less than 5%, thus meeting the requirements of engineering practice.

### 6.2. EMD of AE signal

The original nonstationary AE signal can be decomposed in time and frequency domains using the EMD method and then the first several IMFs with relatively high energy and correlation with the original signal are reconstructed.

**Figure 16:**
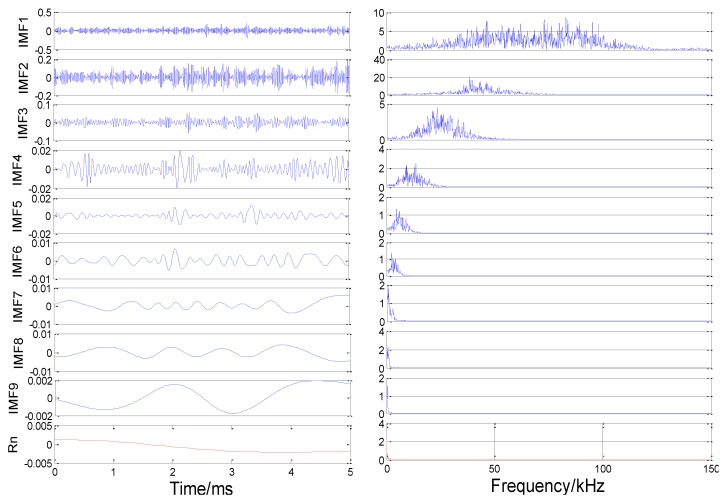
EMD results and spectral diagram of the signal from S1.

Using condition 1 (d1-400, d2-600) as an example, the original AE signal of the pipeline leakage is decomposed using the EMD. Each stage waveform after decomposition and its corresponding frequency spectrum are shown in Figure 16. The original signal is decomposed into nine IMFs and one residual component *R*_n_. In the time domain waveforms, the amplitude of the IMF gradually decreases. Therefore, IMF components with lower signal amplitudes and lower frequencies are negligible and these components are possibly caused by a noisy environment.

The energy of each layer component $E_{MFi}$ and the energy ratio of this layer $R_{IMFi}$ can be obtained to further quantify the IMF using the formula as follows:

(9) $$E_{IMFi}=\frac{1}{N}\sum_{m=0}^{N-1}h_{i}{\left(m\right)}^{2},R_{IMFI}=\frac{E_{IMFi}}{\sum_{i=1}^{9}E_{IMFi}}\times 100\%\;$$

(10) $$R_{IMFI}=\frac{E_{IMFi}}{\sum_{i=1}^{9}E_{IMFi}}\times 100\%\;$$

where $h_{i}\left(m\right)$ is the magnitude of the $i_{th}$ and the IMF component and N is the length of the signal.

**Figure 17:**
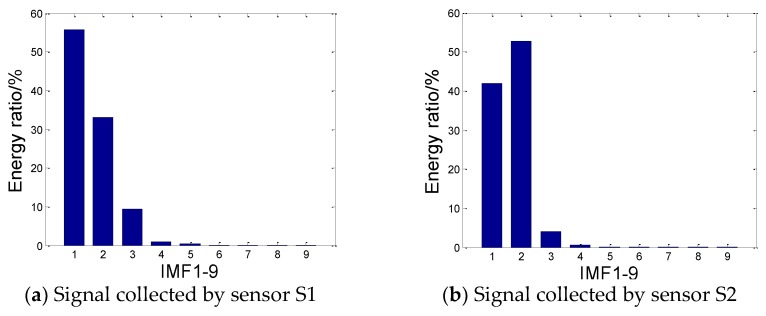
Energy ratio of each IMF component.

The energy ratio of the IMF at various levels is shown in Figure 17. As presented in the figure, the first three IMFs contain almost 98% of the entire signal, which can reflect the main information of the original signal completely. Therefore, the three principal components, that is, IMF1, IMF2 and IMF3, can be used for signal reconstruction, whereas other components with lower energy can be ignored.

**Figure 18:**
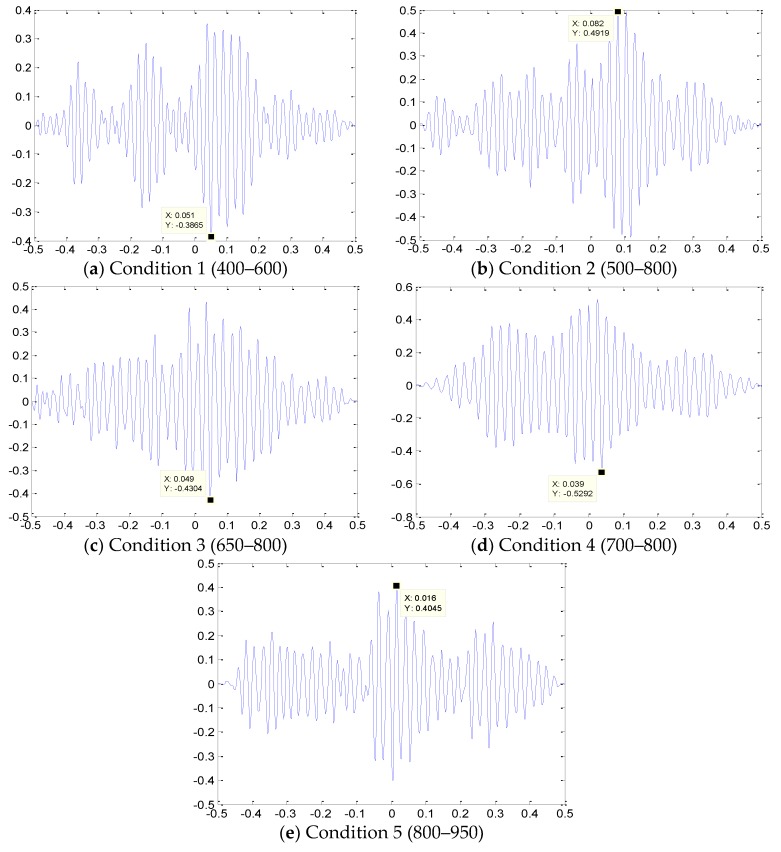
Correlation functions of different conditions and EMD denoising.

The correlation function of the reconstructed waveform is calculated. The cross correlation function diagram under five conditions is shown in Figure 18. The time difference is obtained from the peak value of the figure and the leakage location on the pipe is calculated through the leakage location formula. The positioning results are listed in Table 6.

Based on the results in the table, the original AE signal is decomposed by the EMD, the main IMF reconstruction signal with relatively high energy and large correlation with the original signal is selected and the main IMFs are selected to reconstruct the signal. The leakage source is determined using a time-delay estimation method based on cross correlation. The accuracy of the location calculation is high and the relative error is basically controlled within 5%, thus fully meeting the actual requirements of the project.

### 6.3. Wiener Prefilter and GCC Method

**Figure 19:**
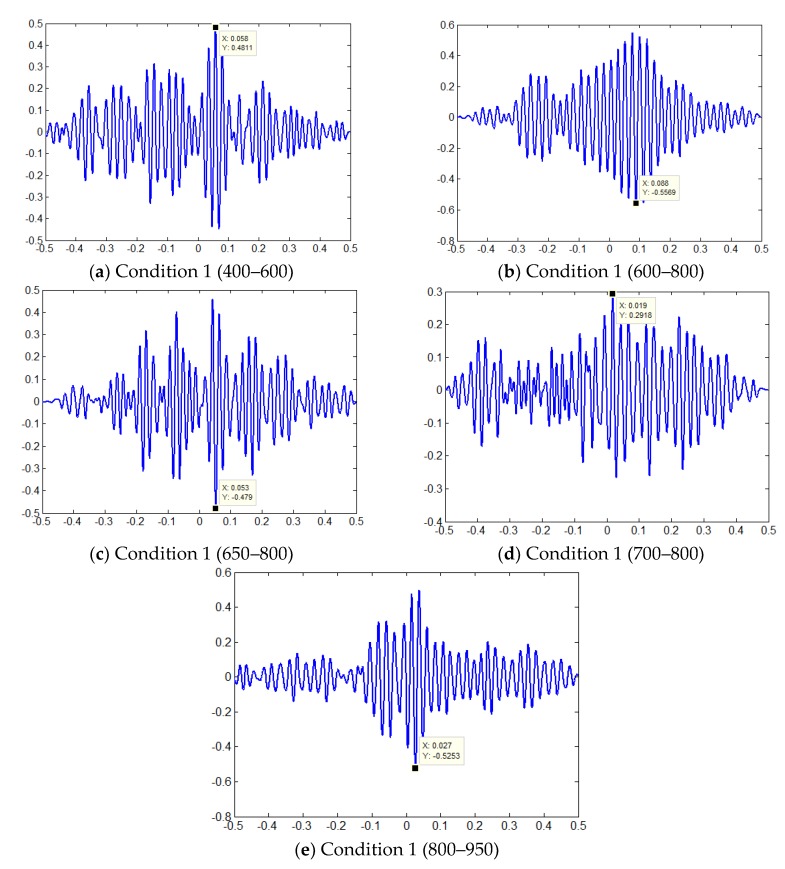
Correlation functions between different conditions and the Wiener pre-filter and GCC method.

The Wiener pre-filters and GCC have been widely used for denoising and leakage location [14]. Figure 19 provides the correlation functions between different conditions and the Wiener pre-filter and GCC method and Table 7 shows the location results of all test conditions. It is clear that the proposed wavelet denoising and EMD denoising process have an accuracy similar to Wiener filtering. The basic cross correlation (BCC) works as well as the GCC.

## 7. Conclusions

In this study, a leakage AE signal is collected via an experiment with sensors inside a liquid-filled pipe. First, detection and recognition of the pipe running status are performed using SVM and AE technology. Then, wavelet decomposition and EMD are used to effectively denoise the original AE signal. Finally, the pipeline leakage source is accurately identified using the cross correlation delay estimation method. This study provides a reference value for a real-time online monitoring of pipeline operational status and has broad application prospects. The main research conclusions are summarized as follows:

(1) In a liquid-filled pipe, the characteristics of the AE signal under different operating conditions vary significantly. The signal is continuous with standing wideband and low frequency, which is a characteristic of a typical noise signal. When liquid flows, a small fluctuation exists in the signal and the frequency spectrum indicates an area of energy concentration, whereas the amplitude is small. When leakage occurs, the signal suddenly presents the typical characteristics of an AE signal with narrowband and noticeable peak frequencies: the energy is concentrated and the amplitude is large.

(2) The SVM method can effectively classify the conditions of pipeline operation with an accuracy rate of more than 95%. The test results show that for inseparable linear problems, the RBF kernel function is mapped to a high-dimensional space for classification and the recognition accuracy evidently improves. Meanwhile, the larger the training dataset, the more accurate the SVM model and the better the classification effect will be.

(3) The propagation speed of the AE wave on the surface of a liquid-filled pipe is 3446 m/s, as determined by the lead-cutting experiment. Both types of noise reduction methods, namely wavelet decomposition and EMD, have ideal denoising ability for original leakage AE signals. The cross correlation time-delay estimation method can be used to accurately locate the pipeline leakage source. The positioning error is basically within 5%, which has certain practical significance in engineering applications.

This article mainly used built-in sensors to detect pipeline leakage. The next phase of our research should include: (1) research with mobile robots and the optimization of robot fixed position, (2) the impact of leakage at different locations on the signal, which requires further study.

## Figures and Tables

**Table 1:** Parameter settings of acoustic emission (AE) instruments.

Hit Length	Threshold	Sampling Rate	Preamplifier Gain	PDT/μs	HDT/μs	HLT/μs
1024	20/35 dB	1 MHz	40 dB	200	800	1000

**Table 2:** Technological parameters of the SR40M sensor.

Size/mm	Temperature/°C	Bandwidth/kHz	Resonant Frequency/kHz	Peak Sensitivity/dB
Φ22 × 36.8	−20–120	15–70	40	>75

**Table 3:** Experimental cases.

Case	Pipeline Status	Pressure/MPa	Sample Number	Distance from Leakage/mm
C1	resting	—	600	400
C2	operational	—	600	400
C3	leakage	0.2	600	400
C4	small pressure leakage	0.05	600	400
C5	large pressure leakage	0.3	600	400

**Table 4:** Case 1: Comparison of the results of locating the leakage source.

Result	d1/mm	d2/mm	Δt/ms	Calculated Value: d1	Absolute Error	Relative Error
Original signal	400	600	0.045	422.47	22.47	5.62%
Wavelet denoising	400	600	0.056	403.51	3.51	0.88%

**Table 5:** Results of locating the leakage source using the wavelet denoising.

Experiment Number	d1/mm	d2/mm	Δt/ms	Calculated Value: d1	Absolute Error	Relative Error
1	400	600	0.056	403.51	3.51	0.88%
2	500	800	0.079	513.88	13.88	2.78%
3	650	800	0.049	640.57	9.43	1.45%
4	700	800	0.017	720.71	20.71	2.96%
5	800	950	0.023	835.37	35.37	4.42%

**Table 6:** Locating results of the leakage source using AE technology.

Experiment Number	d1/mm	d2/mm	Δt/ms	Calculated Value: d1	Absolute Error	Relative Error
1	400	600	0.051	412.13	12.13	3.03%
2	500	800	0.082	508.71	8.71	1.74%
3	650	800	0.049	640.57	9.43	1.45%
4	700	800	0.039	682.80	17.2	2.46%
5	800	950	0.016	847.43	47.43	5.97%

**Table 7:** Results of locating the leakage source using the GCC and Wiener pre-filter.

Experiment Number	d1 (mm)	d2 (mm)	Δt/ms	Calculated Value: d1	Absolute Error	Relative Error
1	400	600	0.058	400.06	0.06	0.015%
2	500	800	0.076	519.05	19.05	3.81%
3	650	800	0.053	633.68	16.32	2.51%
4	700	800	0.017	717.26	17.26	2.47%
5	800	950	0.027	828.48	35.37	3.36%
